# PEOPLE LIVING WITH PARKINSON DISEASE WITH AND WITHOUT FREEZING: DIFFERENCES USING THE NEURO-QOL MEASUREMENT SYSTEM

**DOI:** 10.1093/geroni/igz038.3508

**Published:** 2019-11-08

**Authors:** Kerri Rawson, Ryan Duncan, Tamara DeAngelis, Timothy Nordahl, Jim Cavanaugh, Gammon Earhart, Theresa Ellis

**Affiliations:** 1 Washington University School of Medicine, St. Louis, Missouri, United States; 2 Boston University, Boston, Massachusetts, United States; 3 University of New England, Portland, Maine, United States

## Abstract

Parkinson disease (PD) is the second most common neurodegenerative disease and approximately half of those diagnosed with PD will experience freezing of gait (FOG). FOG is a severe motor disturbance that prevents stepping despite the intention to do so and may be associated with anxiety, decreased cognitive functioning, depression, and poorer quality of life. In this study, we administered the short-form Quality of Life in Neurological Disorders (Neuro-QOL) measurement system to 43 people with PD (28 non-freezers and 15 freezers) and determined freezing status using the New Freezing of Gait-Questionnaire. Freezers had greater motor severity as measured with the MDS-UPDRS-III (p =.003) and poorer balance using the Mini-BESTest (p = .017). Data from the Neuro-QOL battery indicated freezers had greater difficulty with Lower Extremity Function (p = .019) and Upper Extremity Function (p = .027). Freezers also had poorer scores on the Positive Affect and Well-Being (p = .005), Satisfaction with Social Roles and Activities (p = .005), and Stigma (p = .026) scales, but less difficulty on the Communication scale (p = .005) than non-freezers. There were no differences between freezers and non-freezers on the Ability to Participate in Social Roles and Activities, Anxiety, Cognitive Function, Depression, Emotional and Behavioral Dyscontrol, Fatigue, or Sleep Disturbance scales. These findings suggest freezers are less likely to have a positive outlook and satisfaction with their daily lives in combination with poorer functioning. Interventions that target freezing with the potential to improve functioning may result in better quality of life among freezers.

